# The Use of Xanthan Gum as Vaccine Adjuvant: An Evaluation of Immunostimulatory Potential in BALB/c Mice and Cytotoxicity In Vitro

**DOI:** 10.1155/2017/3925024

**Published:** 2017-05-07

**Authors:** Rodrigo Andrade Schuch, Thaís Larré Oliveira, Thaís Farias Collares, Leonardo Garcia Monte, Guilherme Roig Inda, Odir Antonio Dellagostin, Claire Tondo Vendruscolo, Angelita da Silveira Moreira, Daiane Drawanz Hartwig

**Affiliations:** ^1^Programa de Pós-Graduação em Biotecnologia, Centro de Desenvolvimento Tecnológico, Universidade Federal de Pelotas, Pelotas, RS, Brazil; ^2^Centro de Ciências Químicas, Farmacêuticas e de Alimentos, Universidade Federal de Pelotas, Pelotas, RS, Brazil; ^3^Departamento de Microbiologia e Parasitologia, Instituto de Biologia, Universidade Federal de Pelotas, Pelotas, RS, Brazil

## Abstract

The successful production of new, safe, and effective vaccines that generate immunological memory is directly related to adjuvant feature, which is responsible for increasing and/or modulating the immune response. Several compounds display adjuvant activity, including carbohydrates. These compounds play important roles in the immune response, as well as having biocompatible properties in vaccine formulations. One such carbohydrate is xanthan gum, a polysaccharide that is produced by the plant-pathogenic bacterium* Xanthomonas *spp., which has adjuvant attributes. This study evaluated the immune response induced by xanthan gum associated with ovalbumin in BALB/c mice, which were subcutaneously immunized, in terms of antibody production (IgG1, IgG2a, IgG2b, and IgG3), and assessed the levels of IFN-*γ* in the splenocyte culture using indirect ELISA. Furthermore, we investigated in vitro cytotoxicity of xanthan in the embryo fibroblasts cell line of the NIH/3T3 mouse by MTT assay and propidium iodide uptake assay. The mice immunized with ovalbumin plus xanthan gum exhibited higher antibody IgG1 responses than control groups. Furthermore, the xanthan polysaccharide was capable of increasing the immunogenicity of antigens by producing IFN-*γ* and did not exhibit cytotoxicity effects in NIH/3T3 mouse fibroblast cells, considered a promising candidate for vaccine adjuvant.

## 1. Introduction

Vaccine adjuvants are compounds used to improve the immunogenicity of a particular antigen [1]. Aluminum-based mineral salts, approved for human use by the US Food and Drug Administration (FDA), are the most widely used vaccine adjuvants since 1920, inducing predominantly antibody responses. As such, the discovery of new adjuvants is crucial for the development of vaccines that require a cell-mediated response [2, 3]. Modern adjuvant development is based on enhancing and shaping vaccine-induced responses without compromising safety by selectively adding well-defined molecules, formulations, or both [4]. New adjuvant formulations are in advanced stages of development and licensing. Different compounds have demonstrated adjuvant ability, including bacterial products, emulsions, nucleic acids, and microparticles. However, preclinical trials show the lack of basic safety requirements for humans use [5–9].

Numerous polysaccharides originated from plant and microbes have been tested for their potential applications as adjuvants in vaccinations [10]. Each of these carbohydrate-based vaccine adjuvants can be very different from one another and can offer their own physical and chemical characteristics, immunological behavior, and unique attributes. As such, there is a wide range of options available for their use in vaccine development. Furthermore, many of these options have an established history of safety and tolerability due to easy biodegradation and biotransformation [11].

Xanthan gum is a complex extracellular polysaccharide that is produced by the plant-pathogenic bacterium* Xanthomonas *spp., which has viscous properties. Commercial xanthan is widely used as a thickener or viscosifier and a stabilizer in the food and pharmaceutical industry [12]. This polysaccharide consists of a cellulose-based main chain linked to a trisaccharide side chain composite of two D-mannose units with alternating D-glucuronic acid residues. The D-mannose units can be differently acetylated; approximately half the outer D-mannose contains pyruvic acid residues; these anionic substituents influence both the chemical and physical properties of xanthan [13]. The first reports of the intrinsic adjuvant properties of xanthan gum originally emerged in the 1980s [14]. More recently, xanthan has been successfully used in bioadhesive formulations for intranasal influenza virus immunizations [15, 16], and it has been proven capable of enhancing the immunogenicity of recombinant antigens and generating protection against the pathogenic strains of* Leptospira interrogans* [17].

However, the immune response generated by this polysaccharide when employed as vaccine adjuvant has not previously been studied. In the present study, we characterize the immune response elicited by polysaccharide xanthan using a well-characterized model antigen, ovalbumin (OVA), which is a immunogenic antigen that has often been used as a proof of principle for numerous vaccination strategies [18, 19].

## 2. Methods

### 2.1. Animals

Female BALB/c mice (from Central Animal Facility, Federal University of Pelotas, Brazil), aged between five and six weeks, were used in this study. The animals were acclimated for one week before use. Feed and water were offered ad libitum, and the mice were kept in photoperiod for 12/12 hours at 24°C temperature and 50% humidity. All experiments were conducted in accordance with the regulations, policies, and principles of the National Council for Animal Experiments Control in Brazil (CONCEA) and the manual established by the Ethics Committee for Animal Experimentation of the Federal University of Pelotas (UFPel), approved under Protocol number 3418.

### 2.2. Producing the Xanthan Polysaccharide

The* Xanthomonas arboricola* pv.* pruni* strain 106 was used to produce the xanthan gum in a 10 L bioreactor (BioStat B. Braun Biotech International) as previously described [20]. Briefly, the fermented broth was heated at 121°C for 15 min, and the xanthan gum was obtained by precipitation with ethanol [96%, 4 : 1 ratio (v/v)]. The polysaccharide was dried to a constant weight at 56°C and then milled to particle size using a 60–150 mesh. The milled polymer was diluted with ultrapure water (1.25%, w/v) under stirring to provide uniform viscous solution, sterilized, and then stored at 4°C. It was chemically and physically characterized according to viscosity, moisture, ash nitrogen, acetyl, and pyruvate content. Monosaccharides and derivative acids were quantified as previously described [17, 21].

### 2.3. Presence of LPS in the Xanthan

A colorimetric method Limulus Amebocyte Lysate (Pierce, Thermo Scientific) was used according to the manufacturer's instructions to detect the LPS in the aqueous xanthan gum produced. Briefly, a 50 *μ*L standard curve and sample containing different concentrations of aqueous xanthan [0.1–0.5% (w/v)] were dispensed in equilibrated microplates at 37°C in triplicate for 5 min. Then, 50 *μ*L of LAL reagent was added to each well, shacked for 10 seconds, and incubated at 37°C for 10 minutes. For reaction development, 100 *μ*L of substrate solution was added to each well, shacked for 10 s, and then incubated at 37°C for 6 min. The reaction was terminated by the addition of 50 *μ*L of acetic acid 25% [25% (v/v)] and the absorbance measure at 405 nm on a plate reader.

### 2.4. In Vitro Evaluation Cytotoxicity of Xanthan

The effect of the polysaccharide on the viability of the NIH/3T3 mouse fibroblasts cell line was assessed by a colorimetric technique through which the reduction of soluble MTT [3-(4,5-dimethylthiazol-2-yl)-2,5-diphenyltetrazolium bromide] was measured. Cells were seeded at density of 1 × 10^4^ cells per well in a volume of 100 *μ*L in 96-well plates and incubated with different concentrations of an aqueous solution containing the polysaccharide (0.1, 0.25, 0.5 0.75, and 1%), Alhydrogel, or DMSO [1% (v/v)] for 24 and 48 h. The media were removed and 90 *μ*L of medium and 10 *μ*L of MTT (5 mg/mL solution, Sigma) were added to each well. The plates were then incubated for an additional 3 h, and the medium was discarded. The formazan crystals were dissolved by adding 100 *μ*L of DMSO to each well and shaking for 10 min at 200 ×g. The absorbance of each well was read on a microplate reader at 560 nm. The cell inhibitory growth rate (%) was determined as follows: inhibitory rate = (1 − Abs_560  Treated  cells_/Abs_560  control  cells_) × 100%. The data was validated by three independent experiments performed in triplicate.

A propidium iodide (PI) assay was also performed to analyze cellular damage. Briefly, NIT/3T3 cells were seeded at a density of 1 × 10^4^ cells per well in a 24-well plate followed by treatments: xanthan (0.5% w/v), DMSO (1% v/v), Alhydrogel, or medium for 48 h. Following treatment, PI was added (7.5 *μ*M) to each well and left for 15 min and observed by fluorescence microscope using a red excitation wavelength (543 nm) and emission wavelength 615 nm filter under 40x magnification using florescence microscopy (Olympus IX-512, Japan).

### 2.5. Mice Immunization Procedures

**Figure 1:**
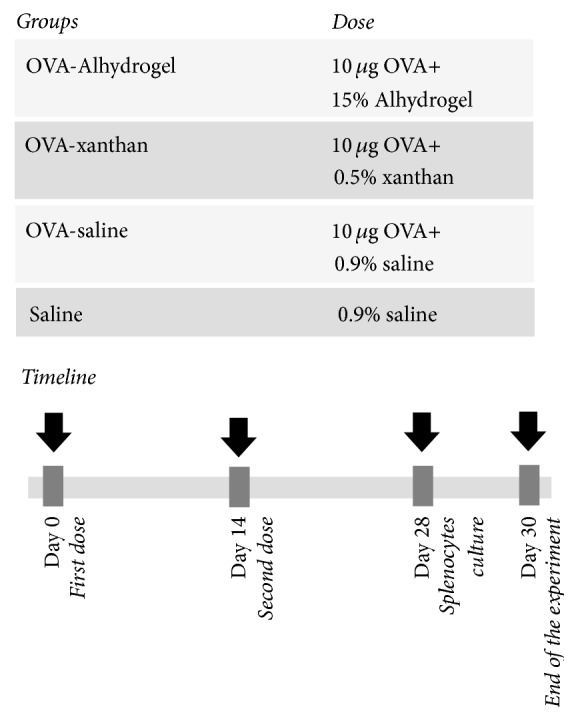
*Schematic representation of vaccination regimen*. Seven mice BALB/c per group receive subcutaneous immunizations with different treatments at intervals of 14 days, followed by splenocytes culture and measurement of IFN-*γ* in culture supernatants and proliferative activity in response to OVA.

Four groups containing seven BALB/c mice were immunized subcutaneously with 10 *μ*g of OVA (InvivoGen, USA) in combination with 15% (v/v) Alhydrogel [2% Al(OH)_3_, InvivoGen, USA], 0.5% (v/v) xanthan polysaccharide, or 0.9% (v/v) endotoxin-free sterile saline (<0.1 EU/mL, InvivoGen, USA). Mice inoculated with a 0.9% (v/v) saline solution were included as a negative control group. The immunizations were performed twice at 14-day intervals, and the animals were euthanized 14 days after the second immunization. The immunization regimen is described in Figure 1. Three independent experiments were conducted. Blood samples were collected from the retro-orbital venous plexus after administration of anesthetic eye drops before each immunization and centrifuged at 5,000 ×g for 10 min. The serum samples were stored at −20°C.

### 2.6. Isotyping of Anti-OVA Antibody Using ELISA

The levels of anti-OVA IgG subclasses in the serum of the mice were determined by indirect enzyme-linked immunosorbent assay (ELISA). The 96-well plates with round bottom wells were coated with OVA diluted in carbonate-bicarbonate buffer, pH 9.6, at a concentration of 100 ng per well for 16 h at 4°C. The ELISA plates were washed three times with PBS-T [PBS with 0.05% (v/v) Tween 20] followed by blocking with 200 *μ*L of PBS-FBS [PBS with 5% (v/v) fetal bovine serum] at 37°C for 2 h. Following three washes with PBS-T, mice serum samples or 0.5% PBS-FBS were added to wells at 1 : 100 in triplicate followed by incubation for 1 h at 37°C before the plates were washed three times with PBS-T. A goat anti-mouse primary antibody isotype IgG1, IgG2a, IgG2b, or IgG3 (Sigma, USA) was added at 1 : 1,000 for 1 h at 37°C, followed by three washes with PBS-T to remove unbound antibodies. For reaction development, peroxidase-conjugated antibody, rabbit anti-goat IgG (Sigma, USA), was added at 1 : 5,000 for 1 h at 37°C and washed five times with PBS-T before the liquid substrate 3,3,5,5′-tetramethylbenzidine (TMB) was added for 15 min at 37°C. The reaction was stopped by adding 2 N H_2_SO_4_/well. The optical density was measured using a microplate reader at 492 nm.

### 2.7. In Vitro Lymphocyte Proliferation Assay

BALB/c mice were euthanized at the end of the immunization protocol, and their spleens were aseptically removed and macerated with a syringe plunger against a fine steel mesh. The erythrocytes were lysed with ammonium chloride [0.9%, (w/v)] and then centrifuged at 300 ×g, 4°C for 10 min. Splenocytes were washed in Hank's Solution and then suspended in DMEM (2 mM L-glutamine, 100 IU/mL penicillin, 100 *μ*g/mL streptomycin, and 50 *μ*g/mL gentamicin) with 10% FBS. The cells were counted after staining with 0.4% trypan blue for viability. Splenocytes were plated at 5 × 10^5^ cells per well in triplicate onto 96-well tissue culture plates (TPP, Sigma). Spleen cells were stimulated with 10 *μ*g/mL of concanavalin A (ConA, positive control); 10 *μ*g/mL of OVA; or medium alone (negative control). Cells were cultured for 48 h at 37°C in 5% CO_2_, and proliferative rates were determined by SRB colorimetric assay. The medium was removed immediately after 48 h, and 100 *μ*L of 10% precooled trichloroacetic acid (TCA) was added per well at 4°C as a fixative. Fixed cells were left on the plate at 4°C for 1 h; then plates were washed five times with distilled water and dried. Cells were stained with 4 mg/mL SRB solution [sulforhodamine B 0.4% (w/v) in 1% acetic acid]; 100 *μ*L was added to each well for 30 min at room temperature, and then the plates were washed five times with a 1% acetic acid solution and dried. The stain was dissolved using 100 *μ*L of 10 mM Tris buffer pH 10.5 and plates were read at 560 nm using UV-visible spectrophotometer. The stimulation index (SI) was calculated as follows: the ratio between the OD (mean) of the cells cultured with the antigen and the OD (mean) of the cells cultured in medium alone in the respective immunized group.

### 2.8. IFN-*γ* in Supernatants of Splenocyte Cultures

Splenocytes were isolated from the immunized mice using the process previously described. The suspensions cells were plated in 24-well tissue culture plates (TPP; Sigma), containing 2 × 10^6^ cells/well. The samples were incubated with OVA (10 *μ*g/mL), ConA (10 *μ*g/mL), or medium, at 37°C in 5% CO_2_. After 48 hours, cell-free culture supernatants were collected and stored at −70°C. Levels of IFN-*γ* were measured by ELISA (BD Bioscience, USA). The capture antibody was added and the plates were incubated for 16–18 h at 4°C. The ELISA plates were washed five times with PBS-T, followed by incubation with blocking buffer [PBS with 10% (v/v) fetal bovine serum] for 2 h at room temperature. A standard concentration curve was created by cytokine dilution (0 to 2,000 pg). Then, 100 *μ*L of each supernatant sample or standard was added and incubated for at least 2 h at room temperature. Subsequently, after five washes with PBS-T, antibody detection solution was added at 1 : 2,000 for 2 h and, following five washes with PBS-T, a working substrate was added to each well. Incubation then proceeded at room temperature for reaction development. The optical density was measured using a microplate reader (405 nm with correction set at 650 nm) in an ELISA plate reader (results expressed in pg/mL).

### 2.9. Statistical Analysis

Data were expressed as mean ± SD and significant differences between groups were determined using analysis of variance (ANOVA) and Tukey's posttest via the Prism 6 software (GraphPad Software Inc., La Jolla, CA, USA).* P* values < 0.05 were considered to be statistically significant.

## 3. Results

### 3.1. Chemical Characterization and Quantification of LPS in the Xanthan Gum

The chemical characterization of xanthan has been previously described [17]. The xanthan gum used in this experiment had moisture, ash, nitrogen, acetyl, pyruvate, and good viscosity in accordance with the recommendations by the FAO and Burlock for xanthan used as food additives [22, 23]. We also investigated the presence of LPS in various concentrations of xanthan gum by LAL endotoxin test. All concentrations tested exhibited low amounts of endotoxins (<0.2 EU/mL) (data not show).

### 3.2. Cytotoxicity of Xanthan Gum

**Figure 2:**
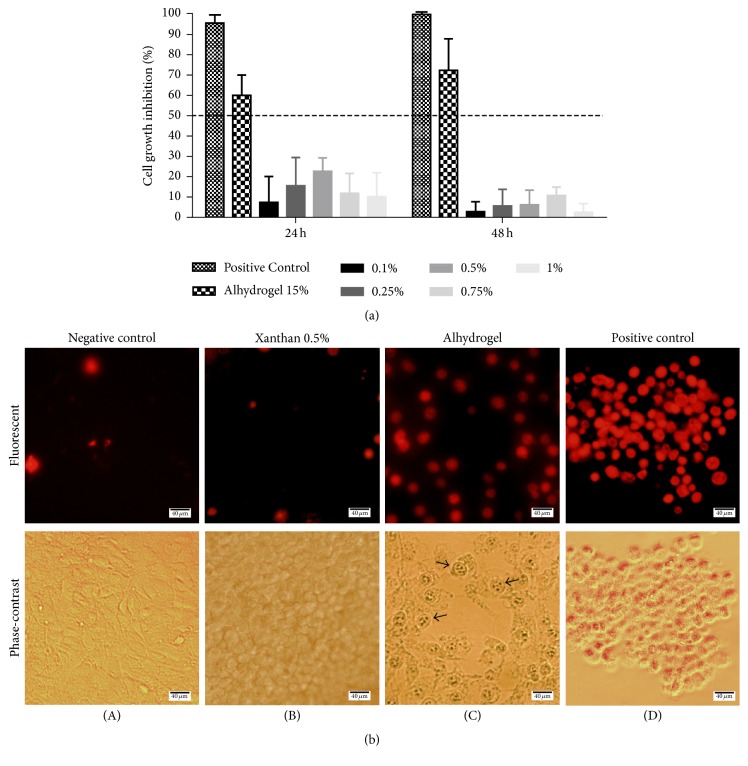
*In vitro cytotoxicity of the aqueous xanthan solution*. (a) The effect of different concentrations of aqueous xanthan solutions on the inhibition of NIH/3T3 cells was determined using an MTT assay. The inhibition rate was related to the negative control (DMEM). Cells were treated with DMSO 1% as positive control. The data are expressed as the means ± SD of three independent experiments. (b) Representative composite images show cell viability by propidium iodide (PI) staining after the cells were treated with xanthan 0.5% (B) or Alhydrogel 15% (C) for 48 hours, stained with PI, and visualized by fluorescent and phase-contrast microscopy. Untreated cells (A) and those treated with DMSO 1% (D) as controls of experiments. Red fluorescence indicates membrane damage and PI uptake. Arrows represent dead cells with apoptotic nuclei. Original magnification ×40.

The antiproliferative activity of xanthan was studied in NIH/3T3 mouse fibroblasts cell line. The cell viability was determined by MTT assay after 24 and 48 h of incubation with different concentrations of xanthan (Figure 2(a)). Cells incubated with medium only were taken as negative control, while those incubated with DMSO (1.0%) were the positive control. None of the aqueous xanthan solutions (0.1–1.0%) were cytotoxic in vitro. No statistically significant differences in the growth rate (<50%) were observed between the different xanthan concentrations and the negative controls over the 24 or 48 h period (*P* > 0.05). In contrast, Alhydrogel exhibited growth inhibition rates above 50%, demonstrating cytotoxic effect in fibroblasts cells.

The effects of the adjuvant formulations were also tested to assess the change in cell morphology after 48 h (Figure 2(b)). Red-fluorescent propidium iodide is a cell impairment DNA-binding dye that indicates an increase in plasma membrane permeability and loss of plasma membrane integrity. Both the cells treated with xanthan and the untreated cells exhibited low red fluorescence (Figure 2(b), (A) and (B)), which denoted regular and intact cells. This was confirmed by phase-contrast image. A change in the morphology of the cells treated with xanthan was also identified when compared to the nontreated cells. Observation of the cells treated with Alhydrogel 15% (Figure 2(b), (C)) identified damaged cellular membrane with uptake by PI, with morphological alterations when compared to the untreated control. These cells also exhibited typical features of apoptosis such as apoptotic nuclei with highly condensed or fragmented chromatin in multiple sites.

### 3.3. Humoral Immune Response in Immunized Mice

**Figure 3:**
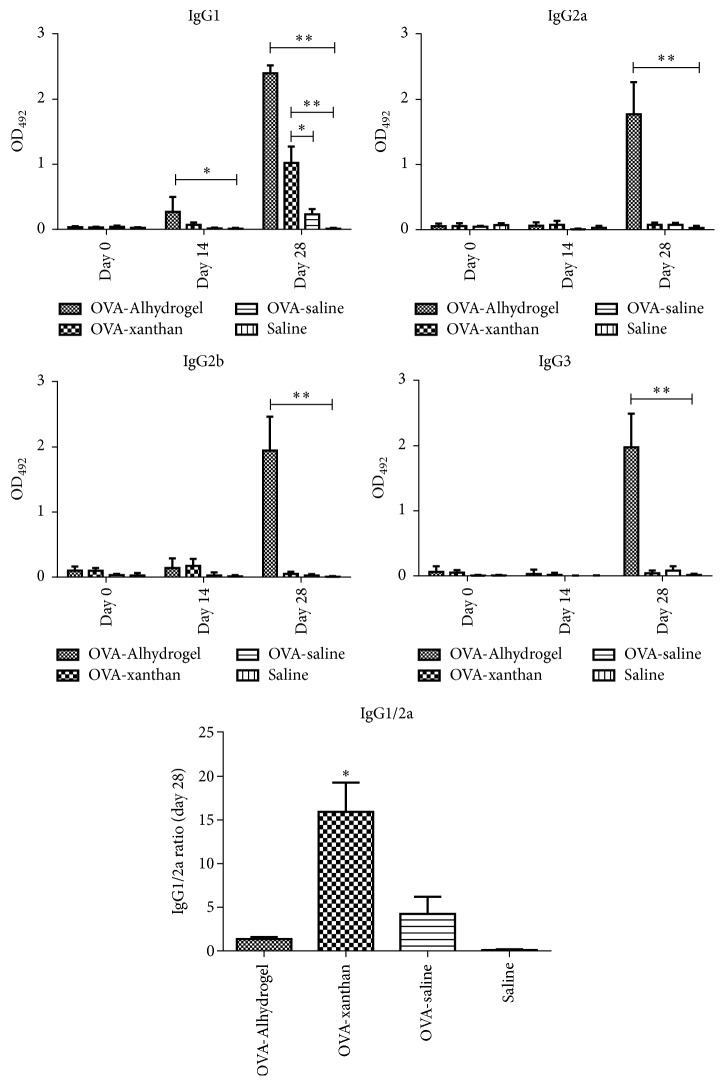
*IgG Isotypes antibodies induced by xanthan gum*. BALB/c mice were subcutaneously immunized twice at a two-week interval with OVA (10 *μ*g) alone as a control or in combination with Alhydrogel (15%) or xanthan (0.5%). A group immunized with saline solution was used as control. Blood was collected on days 0, 14, and 28 after immunization. The responses of the IgG isotypes (IgG1, IgG2a, IgG2b, and IgG3) and IgG1/2a ratio at day 28 were determined by ELISA with serum dilution 1 : 100 and humoral response compared to the control group (OVA-saline). The values shown are the mean ± SD of three independent experiments. (^*∗*^*P* < 0.05, ^*∗∗*^*P* < 0.005).

In order to assess the specific antibody response in groups of mice immunized with OVA, blood samples were collected on days 0, 14, and 28, and the levels of the subclasses of IgG (IgG1, IgG2a, IgG2b, and IgG3) present in the serum were determined by ELISA (Figure 3). The group OVA-xanthan was able to induce humoral response mediated by IgG1 after the second immunization (day 28), and this was higher than the ovalbumin-saline group (*P* < 0.05). IgG1 antibody levels were also detected after the first immunization (day 14) and second immunization (day 28) for the OVA-Alhydrogel group. For the other IgG subclasses, only the OVA-Alhydrogel group had detectable humoral immune response (*P* < 0.05) after the second immunization. The other groups exhibited no statistically significant levels of antibodies to IgG2a, IgG2b, or IgG3.

### 3.4. Splenocyte Proliferation Evaluated by SRB

**Figure 4:**
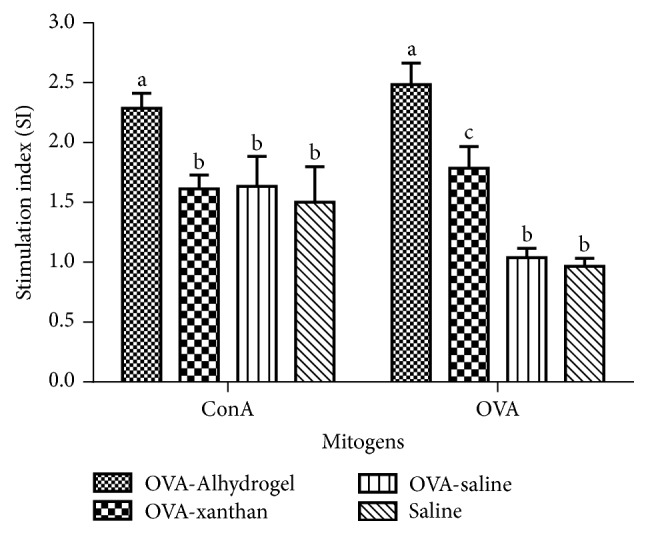
*Splenocytes proliferative responses*. Splenocytes were prepared two weeks after the last immunization and cultured for 48 h with ConA (final concentration 10 *μ*g/mL) or OVA (final concentration 10 *μ*g/mL). The proliferation of splenocytes was measured using the SRB method as described and shown as a stimulation index (SI). The values represent mean ± SD of three independent experiments (*n* = 7 per group). Bars with different letters represent *P* < 0.05.

The effects of xanthan on splenocyte proliferative responses to ConA and OVA stimulation were evaluated by SRB assay (Figure 4). Splenocyte obtained from group OVA-xanthan (stimulation index = 1.78) and OVA-Alhydrogel (SI = 2.42) stimulated ex vivo with OVA proliferated at higher levels than those in the OVA-saline group (Figure 4). The saline group (negative control) did not stimulate splenocyte, thus demonstrating that the CD4 T-cell lymphoproliferation was a specific response to OVA. The addition of OVA to lymphocytes from animals that had not been primed with the antigen produced nonsignificant levels of proliferation. Cells were treated with ConA, which was employed as a positive control for the experiment.

### 3.5. Production of IFN-*γ* by Splenocyte Culture

**Figure 5:**
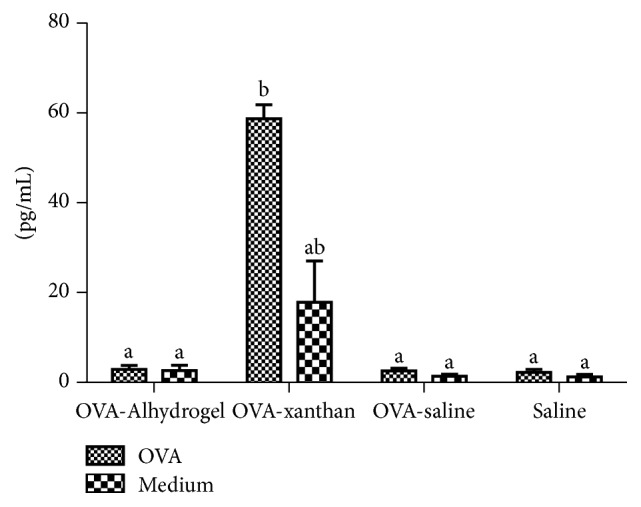
*Quantification of IFN-γ in splenocytes culture by ELISA*. Spleen cells were isolated and cultured in 24-well tissue culture plates (5 × 10^6^ cells/well) for 48 h. The levels of the IFN-*γ* in cell-free culture supernatants were measured by ELISA. The values represent the mean ± SD of the three independents experiments. For statistical analysis, concentration values for the immunized groups treated with the OVA were compared with those for the OVA-saline immunized group treated with OVA, and bars with different letters represent *P* < 0.05.

The presence of the cytokine IFN-*γ* was analyzed in supernatants of cultured spleen cells from immunized mice to evaluate cellular response in polarization of immune response. OVA-xanthan immunized mice had higher levels of IFN-*γ* following ex vivo stimulation with OVA than the other groups (Figure 5). The levels of IFN-*γ* were similar to those for the ConA positive control (not shown). In contrast, no IFN-*γ* production was detected when spleen cells from other groups under the same experimental conditions were compared. Measurements of spleen cells from negative control animals stimulated with OVA produced negligible results. These data indicated that the CD4^+^ T-cell responses following immunization with OVA-xanthan were biased toward Th1.

## 4. Discussion

Most licensed vaccines for use in humans work through protective antibody responses mediated by B-cells. This fact has limited the success of vaccines against pathogens whose response is T-cell-dependent. Thus, there is a need for new adjuvants that deliver antigens in a manner as to induce an appropriate T-cell-mediated response [9, 24]. In addition, an appropriate polarization of the immune response is associated not only with the delivery system but also with the nature of the antigen and characteristics of the route and frequency of administration [25]. The correct adjuvant in combination with the antigen should be capable of inducing both humoral and cellular immune responses in vaccines [1]. Xanthan gum, a widely used suspending and thickening agent, has a backbone chain that consists of (1,4) *β*-D-glucan cellulose and is a negatively charged polymer with intrinsic adjuvanticity that is able to activate polyclonal lymphocytes B in the absence of T-cells [14]. However, the biological properties and the mechanism of xanthan adjuvanticity are not clear and, until recently, they have remained unexplored.

A low incidence of adverse events is critical for the advancement of vaccine candidates. The biosafety of xanthan gum is evident in the fact it has been used as an FDA-approved food additive and rheology modifier since 1969 [10, 11]. The parameters evaluated on the constitution of the xanthan produced in this study were within the required standards established by the FAO [22]. However, parameters for xanthan composition as a vaccine adjuvant have yet to be established. To further confirm that the immune response induction by xanthan gum was not mediated by contaminating the LPS, we measured endotoxin contamination in samples of different concentrations of xanthan gum. All concentrations tested contained low amounts of LPS (<0.2 EU/mL) (data not shown), indicating that the antigenicity of xanthan is unlikely to be related to the LPS present in the polysaccharide preparation.

Our study showed that xanthan gum produced from* Xanthomonas arboricola* pv.* pruni* 106 did not affect NIH/3T3 cell viability. All concentrations tested, including the highest, also had no effect on cell viability, highlighting the biocompatibility of the tested polysaccharide. Furthermore, previous studies have demonstrated that xanthan gum preparations have a record of safety in vitro [15, 26] and in preclinical trials [27], confirming the data obtained in this study. In addition, xanthan is regularly consumed by humans without ill effect. Although further testing is required, these findings are encouraging for the clinical development of xanthan as adjuvant vaccine platform.

Most licensed adjuvants work by promoting protective antibody responses. The most abundant immunoglobulin in serum is IgG, which have four subtypes in mice (IgG1, IgG2a, IgG2b, and IgG3). Each of these subtypes has a specific function and affinity to Fc*γ*R receptors on innate immunity cells [28]. Further to their well-defined roles in triggering the activation of innate effector cells, these receptors are important in antigen presentation and maturation mediated by immune complexes on dendritic cells as well as the adjustment and maintenance of B-cell survival [29]. In present study, an antibody response was observed following vaccination with OVA-xanthan. Detectable levels occurred following the administration of the priming dose, and the titers increased with each subsequent boost. Previous work has demonstrated that xanthan gum is able to induce class IgA and IgG antibodies when administrated via mucosal [16]. Data published by our group showed that xanthan gum is able to induce high titers of IgG antibodies when administered subcutaneously with antigen LigANI from* Leptospira interrogans* [17]. This difference may be related to the different antigens and the different animal models studied. In addition, whereas mice that received OVA in Alhydrogel mounted a strong IgG response, mice vaccinated with OVA-xanthan exhibited only IgG1 response. This can be attributed to the mechanism of action of aluminum salts through Th2 immune response, which induced the B-cell's production of antibodies, confirming the data obtained during this experiment [30].

On the other hand, splenocyte proliferation is a crucial event in the activation cascade of both cellular and humoral immune responses [31, 32]. As such, the mitogen effects of OVA on the proliferations of splenocytes were investigated within this study. We demonstrated that splenocytes isolated from animals immunized with OVA plus xanthan are capable of responding to antigens by promoting the proliferation of lymphocytes. OVA would be expected to be preferentially presented by the MHC-II pathway rather than cross-presented by the MHC-I pathway [33–36], which indicates a T CD4+ response in cellular culture.

In addition to B-cell humoral response, we found that xanthan gum increases the immunogenicity of model antigen through cellular responses to IFN-*γ*. This aspect of immune responses is crucial factor in the development of new vaccine adjuvants [8]. A previous study found that xanthan gum was capable of enhancing antitumoral activity in mice by toll-like receptor- (TLR-) 4 recognition, which induces the production of inflammatory cytokines, including TNF-*α* and IL-12 p40, via nuclear factor-*κ*B (NF*κ*B) [37]. In addition, most of the similar carbohydrate-based adjuvants, such as lentinan, zymosan, mannan, and muramyl dipeptide (MDP), which act by binding to the innate immune response receptors that recognize pathogen-associated molecular patterns (PAMPs), including TLRs, NOD2, and C-type lectins, result in proinflammatory cytokine production [10, 24, 38]. Furthermore, *β*-1,3-D-glucan polymers purified from* Saccharomyces cerevisiae* have demonstrated the ability to induce IgG1 and IgG2c antibodies and T-cell responses Th1 to IFN-*γ* in mice [24]. In combination with proinflammatory cytokines, TLR-induced signal(s) is/are required for memory CD4+ T-cell differentiation (but not for the activation of memory T-cells), inducing dendritic cells maturation and migration to the lymph nodes, as well Th1 induction [39, 40]. Thus, the xanthan gum has immunostimulatory capabilities that make it a great adjuvant for future vaccine formulations.

## 5. Conclusion

In the present study, we demonstrated the adjuvant effect of xanthan gum when used with OVA. Summarily, it was able to stimulate cellular immune response by cytokine IFN-*γ* and humoral response via IgG1. Furthermore, the xanthan polysaccharide was considered to be biocompatible when tested in vitro. These results show the use of xanthan gum as a promising vaccine adjuvant.
